# RMHIL: A Rule Matching Algorithm Based on Heterogeneous Integrated Learning in Software Defined Network

**DOI:** 10.3390/s22134739

**Published:** 2022-06-23

**Authors:** Yiping Guo, Guyu Hu, Dongsheng Shao

**Affiliations:** 1 Command and Control Engineering College, People’s Liberation Army Engineering University, Nanjing 210007, China; huguyu@aeu.edu.cn; 2 Unit 31106 of People’s Liberation Army, Nanjing 210007, China; s2609529342@163.com

**Keywords:** SDN, flow forwarding, rule matching, heterogeneous integrated learning

## Abstract

To ensure the efficient operation of large-scale networks, the flow scheduling in the software defined network (SDN) requires the matching time and memory overhead of rule matching to be as low as possible. To meet the requirement, we solve the rule matching problem by integrating machine learning methods, including recurrent neural networks, reinforcement learning, and decision trees. We first describe the SDN rule matching problem and transform it into a heterogeneous integrated learning problem. Then, we design and implement an SDN flow forwarding rule matching algorithm based on heterogeneous integrated learning, referred to as RMHIL. Finally, we compare RMHIL with two existing algorithms, and the comparative experimental results show that RMHIL has advantages in matching time and memory overhead.

## 1. Introduction

With the continuous expansion of network scale and the rapidly changing business environment of the Internet, the drawbacks of traditional networks have gradually become apparent. First of all, the flexibility and agility of the network urgently need to be improved [1]. Secondly, network delays and operational costs pose challenges to the efficiency of network operations [2].

In 2006, SDN was born in the Clean Slate project of Stanford University in the United States. To effectively solve the shortcomings of traditional IP networks in maintenance, expansion, and experimental innovation, the team led by Professor Nick McKeown proposed to bring programmable features to the network based on Openflow, and the software defined network (SDN) came into being [3]. In 2009, Professor Mckeown formally proposed SDN, a new network architecture in reference [4], breaking the traditional closed mode of software and hardware integration and separating the control level of network equipment from the data forwarding level.

Compared with traditional networks, SDN has the following advantages: (1) Flexibility; as it solves the problems caused by manual equipment configuration in traditional networks and allows the configuration of network equipment to be dynamically adjusted according to needs. SDN separates the control rights of network equipment and manages them by a centralized controller. There is no need to rely on the underlying network equipment, and the differences from the underlying network equipment are shielded. The equipment configuration can be flexibly implemented at the control layer. (2) Scalability; the decoupling of hardware and business in SDN provides scalability for SDN [5]. Compared with traditional networks, the correlation between hardware and business in SDN is greatly weakened. Users can customize any network routing and transmission strategy they want to meet their business requirements [6], while the network hardware only needs to process and forward data as usual. SDN can carry customizable business requirements, meaning it is scalable. (3) High efficiency; the introduction of centralized controllers provides a global-oriented strategy deployment for the network. At the same time, the network can realize automatic deployment, operation, maintenance [7], and fault diagnosis, which reduces the delay of network management and the cost of network operation and maintenance to some extent.

Carrying out flow scheduling in SDN can provide a real-time global network view [8] and flexible control methods [9] for network measurement and management, which have outstanding research significance.

SDN flow scheduling is divided into the control layer and data layer. We focus on data layer flow scheduling. Data layer flow scheduling means that flows are forwarded between data layer switches (that is, switches that only have the forwarding function) according to the rules issued by the control layer. The specific process is as follows: when a flow reaches a switch in SDN, if the flow matches a rule in the flow table of the switch, it will be forwarded according to the matched rule; if the flow does not match any rule in the flow table of the switch, its first packet will be sent by the switch to the SDN control layer. After receiving the data packet, the control layer calculates the forwarding path of the corresponding flow in the network and issues new rules to each switch on the forwarding path. The switch updates its own flow table accordingly. Subsequent packets of this flow and all other flows that can match this rule are forwarded according to this rule without the intervention of the control layer [9].

In data layer flow scheduling, rule matching occupies an important position. The matching performance directly affects the forwarding efficiency of the flow, which affects the overall flow scheduling efficiency. The flow forwarding time is an important evaluation indicator for flow scheduling [10]. In the face of large-scale networks, huge traffic pressure puts forward higher requirements on the flow forwarding time. If the time overhead of rule matching can be reduced, the overall flow forwarding time will be greatly reduced. At the same time, in the process of flow forwarding, rule matching is a necessary step executed multiple times. If the memory overhead of rule matching can be reduced, the storage pressure on overall flow scheduling will be greatly reduced.

Therefore, this article will research the rule matching algorithm for SDN flow forwarding to reduce matching time and memory overhead.

The existing solution to the matching problem of forwarding rules relies on artificially adjusted heuristic algorithms to build decision trees. This type of solution has limitations. 

(1) It faces the trade-off between algorithm performance and overhead. References [11,12,13] adopt the most intuitive solution, which is to traverse the rules. Reference [11] proposes the recursive and non-recursive algorithms for traversing binary trees. Reference [12] proposes a graph depth-first search traversal algorithm. Reference [13] proposes a rule matching algorithm based on post-root skip traversal. Such “dumb” traversal algorithms have no advantage in time or cost. References [14,15] propose building decision trees for rule sets. Reference [14] proposes an intelligent hierarchical decision tree construction algorithm. Reference [15] proposes an algorithm called HyperCuts; it shows that the existence of range rules makes the rules in the rule set may overlap each other in certain domains. Such algorithms take relatively little time but require significant overhead. When building a decision tree, there will inevitably be a problem of rule overlap, which will cause serious storage space consumption and some time loss. 

(2) The existing algorithms [11,12,13,14,15,16,17,18] do not directly optimize the global goal. The local goal of each step is poorly related to the global goal, making it difficult to obtain the optimal decision tree for the global goal. A good rule matching algorithm should reduce matching time and memory overhead as the global goal. References [16,17,18] solve the overlapping rule problem, but they are difficult to guarantee the global optimality of the final solution. Reference [16] proposes a rule matching algorithm, EffiCuts, that non-uniformly partitions the rule space. Reference [17] proposes a rule matching algorithm CutSplit that combines Cutting and Splitting. Reference [18] proposes an algorithm called CutTSS. Even though CutTSS is presently one of the best performing algorithms, it is still necessary to use greedy strategies when building decision trees. 

(3) Facing the large-scale flow table of SDN, the number of flow table entries and fields used for rule matching is huge, making it difficult for such schemes to effectively construct a decision tree. An architectural approach based on Ternary Content Addressable Memory (TCAM) has been the dominant implementation of rule matching in the industry. With the deployment of SDN-based applications, the number of rule fields has increased dramatically, outpacing the evolution of TCAM capacity. For example, OpenFlow1.3 Switch checks more than 40 fields to complete rule matching, a number expected to grow in the future [19].

Machine learning methods can effectively solve the above limitations. There are two ways to apply machine learning to the forwarding rule matching problem. The first method is direct application [20,21,22,23], replacing the decision tree with a neural network. Inputting a flow packet will output matching rules. Although this end-to-end solution is attractive and the matching speed may be greatly improved, it has two major shortcomings: Firstly, it cannot guarantee that the correct rules will always be matched. Secondly, a large rule set requires a corresponding large neural network model, which may be difficult to complete under general experimental conditions. The second method is heterogeneous integration, which combines heterogeneous learners such as deep learning, reinforcement learning, and decision trees to complete the rule matching task of flow forwarding. We use deep learning and reinforcement learning to build decision trees and search for rules that match the flow according to the constructed decision trees. Deep learning is suitable for large rule sets [24], ensuring good matching results. Reinforcement learning is a method for solving problems with global goals. Deep learning and reinforcement learning are used to build decision trees, and the decision tree for rule matching provides perfect accuracy. 

The rule matching problem for SDN flow forwarding requires that the matching results have one hundred percent accuracy. Therefore, we adopt the second method.

We find that the heterogeneous integrated learning algorithm can be applied to the rule matching problem in the process of SDN flow forwarding. Through the integration of machine learning methods such as recurrent neural network (a type of deep learning algorithm), reinforcement learning, and decision tree, a decision tree dedicated to rule matching problems is constructed. Such a decision tree can meet the one hundred percent accuracy [25] required by the SDN flow forwarding rule matching problem and the low matching time and low memory overhead required by large-scale flow tables.

The main research work is as follows: (1) We describe the SDN flow forwarding rule matching problem and transform it into a heterogeneous integrated learning problem. That is, through the integration of recurrent neural networks, reinforcement learning, and decision tree to learn to construct a decision tree for rule matching. (2) We design and implement an SDN rule matching algorithm based on heterogeneous integrated learning, referred to as RMHIL. (3) We compare RMHIL with two existing algorithms, and analyze the experimental results using the matching time and memory overhead as the benchmark metrics.

## 2. Problem Description and Transformation

### 2.1. Problem Description

A flow is defined as a unidirectional packet flow transmitted between a source IP address and a destination IP address. All packets have a common transport layer source and destination port number [26]. The SDN switch maintains a local flow table for storing rule sets. When the first packet of a flow arrives at the switch, if it can find a matching rule in the switch’s rule set, the rule will be output and the corresponding action will be performed. If not, the packet is sent to the controller. The controller calculates the new rules and sends them to the switch. All subsequent packets of this flow can be matched with the rules in the switch.

This problem can be simplified. Input a packet of a flow, match it with the rule set in the SDN switch (the matching method used in this article is to construct a decision tree of the rule set, and then query the data packet according to the constructed decision tree), output matching rules or negative values (a negative value means that no matching rules were found).

**Figure 1:**
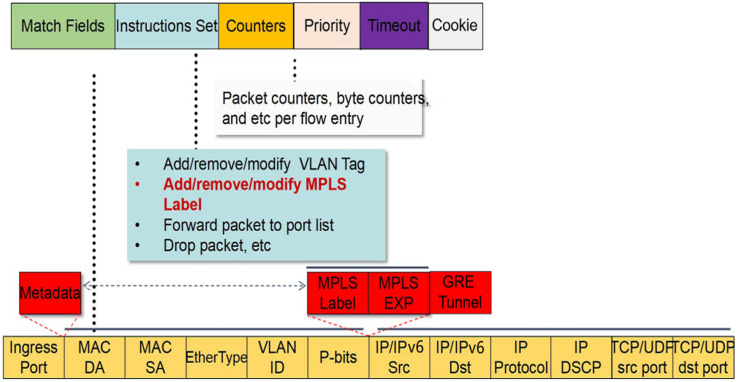
OpenFlow1.3 flow table structure.

Figure 1 shows the information contained in a flow table, including match fields, instructions set, counters, priority, timeout, cookie, and other information. Among them, 40 match fields are used for rule matching. Generally, the fields used for the rule matching of the SDN switch include 5 match fields, including source and destination IP/IPv6 address, TCP/UDP source and destination port number, and protocol. A packet matches a rule if and only if all packet information matches the matching domain information in the rule. Since a packet may match multiple rules, we stipulate that in this case, the packet matches the rule with the highest priority.

For example, Table 1 gives a simple example of a rule set. A rule includes source IP/IPv6 address, destination IP/IPv6 address, TCP/UDP source port number, TCP/UDP destination port number, protocol, instructions set, counters, priority, timeout, cookie, and other fields. We have a packet [source IP/IPv6 address, destination IP/IPv6 address, TCP/UDP source port number, TCP/UDP destination port number, protocol] = [1.0.0.2/32, 1.0.1.2/32, 0, 20, TCP]. In the rule set, two rules with priorities 1 and 2 are successfully matched, and the third rule with the highest priority is taken as the matching result of the given packet.

### 2.2. Problem Transformation

The goal of SDN flow forwarding rule matching is to find the rules that match the flow packet. The step of using heterogeneous integrated learning to solve the rule matching problem is to construct a decision tree based on reinforcement learning and recurrent neural networks, and then search for rules matching the flow according to the constructed decision tree. The following demonstrates that reinforcement learning and recurrent neural networks are suitable for decision tree construction and complete the integrated construction of heterogeneous learners.

Two characteristics of reinforcement learning are very suitable for the decision tree construction problem of the rule set. One is that the return is sparse [27], and there is not necessarily a return every time an action is executed. The goal of reinforcement learning is to optimize the return and not care about the impact of intermediate actions on the return. Similarly, the decision tree of the rule set cares only about the final matching time and memory overhead, not necessarily knowing the impact of intermediate nodes on the final performance. The other is that the rewards of reinforcement learning can be delayed [27]. It would be best to wait for learning to end to understand the rewards and then give feedback. Similarly, the decision tree needs to be constructed before the matching time, and memory overhead can be reported. In addition, as a deep learning algorithm, a recurrent neural network requires many samples. The samples here need to know a series of actions, construction time, and cost for building a decision tree. Since we can use existing software in parallel to build the decision tree of the rule set, we can generate a large number of samples efficiently at low cost.

The above characteristics prove that the decision tree construction of the rule set is suitable for reinforcement learning and recurrent neural network. The following construction transforms the SDN stream forwarding rule matching problem into a heterogeneous integrated learning problem.

**Figure 2:**
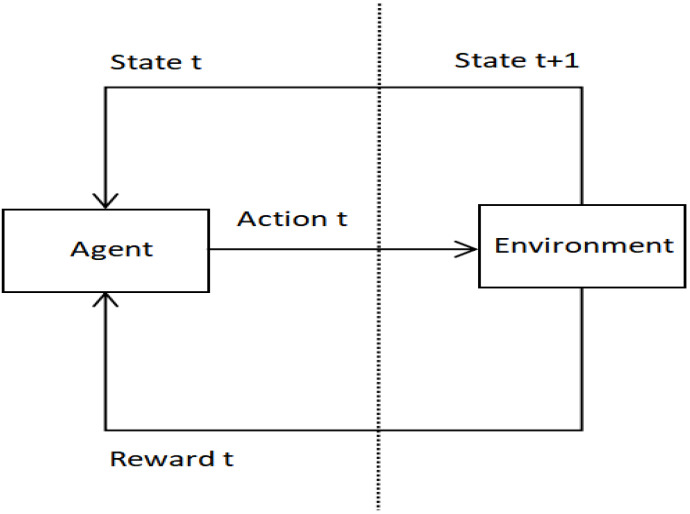
This is a reinforcement learning system, where the final output is given by the agent.

As shown in Figure 2, a reinforcement learning system consists of an agent and a constantly interacting environment. The agent obtains the state t provided by the environment, takes the tentative action t that may change the state (that is, state t + 1) of the environment, and then receives feedback (that is, reward t) from the environment. The agent’s goal is to optimize the return, and the final output is the optimal mapping strategy from state to action.

The description of reinforcement learning tasks usually uses Markov Decision Process (MDP) quadruples E=<X,A,P,R>, where E is the environment, X is the state space, A is the action space, P is the probability of the environment transferring from the current state to another state X×A×X→(0,1), and R is the return.

The environment of the reinforcement learning system is constructed as a rule set and a decision tree. The state x of the environment is the current decision tree. The action a is to divide the node (the node is divided into several sub-nodes along the matching domain fields of the rule set). The reward r is a combination of matching time and memory overhead. By constantly trying to execute actions, we get the strategy π (a series of actions to build a decision tree), and we can know the actions to be executed a=π (x) in the state x. The pros and cons of the strategy depend on the cumulative return obtained after the long-term execution. Finally, the agent selects the strategy with the best cumulative return.

**Figure 3:**
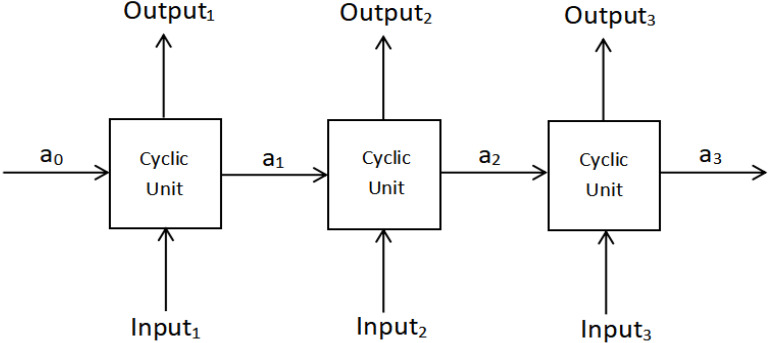
This is a recurrent neural network model, where a is the parameter passed between the cyclic units.

We set the agent as a recurrent neural network model. As shown in Figure 3, parameters are passed between the cyclic units, and the adjusted parameters of the previous unit act on the next unit. In each cyclic unit, we need to input sample (x,y), and the parameters are adjusted by the output $\hat{y}$ of sample x in the neural network. In the agent, sample x is the decision tree state and action, the sample y is the return, and the output $\hat{y}$ of sample x in the model is the return provided by the environment. When the model training is completed (model parameters are determined), the agent (model) can give the optimal mapping strategy from the current decision tree state to the partition nodes.

At this point, the matching problem of SDN flow forwarding rules has been transformed into a heterogeneous integrated learning problem.

## 3. Algorithm Design and Implementation

### 3.1. Algorithm Design

The algorithm in this paper is designed to construct a decision tree of rule sets based on reinforcement learning and recurrent neural networks, and then search for rules matching the flow according to the constructed decision tree. Input a flow packet, a set of rules, and several targets (matching time and memory overhead), learn to build a decision tree for the rule set to minimize the target, and finally output the rules that match the flow packet.

The learning framework proposed in Section 2.2 is formalized as a learning task E=<X,A,P,R>. At each step t, the current state is $x_{t}\in X$ (i.e., the current node of the decision tree), the agent executes action a∈A (i.e., divide nodes) and gets a reward $r_{t}\in R\;$(i.e., matching time and memory overhead). The state transition function P defines that the environment transitions from the current state $x_{t}\;$to the next state $x_{t+1}$ (i.e., the updated node of the decision tree) with a certain probability. The goal of the agent is to maximize the cumulative return $\left[\sum_{t=0}^{+\infty }\gamma^{t}r_{t+1}|x_{0}=x\right]$, where γ is the discount parameter (set as 1 in this article), the initial state $x_{0}\in X\;$is a random decision tree composed of a single node.

For the division of nodes involved in the method, we adopt the approach of most existing algorithms: split the nodes in the decision tree along one or more dimensions. The root node contains all the rules, and the nodes are divided iteratively from the root until each leaf node contains rules fewer than the predefined number.

The problem that needs to be addressed is how to deal with sparse and delayed returns in gradually constructing a decision tree. In theory, rewards can only be given after each tree is constructed, but in fact, we need to provide feedback at every step. Therefore, the node state value function reflecting the cumulative return is designed as follows.

Given a tree node n, $t_{n}$ and $m_{n}$ respectively represent the matching time and memory overhead of node n, $T_{n\;}$and $M_{n\;}$ respectively represent the matching time and memory overhead of the subtree rooted at n.

(1) $$T_{n}=t_{n}+\underset{i\in \mathrm{children}\left(n\right)}{\max}T_{i}$$

(2) $$M_{n}=m_{n}+\sum_{i\in \mathrm{children}\left(n\right)}M_{i}$$

$V_{n}\;$is a value function used to judge the pros and cons of a decision, which represents the cumulative return from the tree node n. It is defined as Equation (3).

(3) $$V_{n}=\lambda \cdot T_{n}+\left(1-\lambda \right)\cdot M_{n}$$

In formula (3), λ is the trade-off coefficient between matching time and memory overhead, λ∈[0,1].

The action a∈A on this node only needs to make the best decision to optimize the matching time and memory overhead of the subtree rooted at this node. We obtain the action a when $V_{n}=\lambda \cdot T_{n}+\left(1-\lambda \right)\cdot M_{n}$ is minimized. The return maximization in this section is the minimization of matching time and memory overhead.

The values of the above formulas need to be generated after the construction of the decision tree is completed.

The flow of the learning decision tree construction strategy is shown in Algorithm 1 (hereinafter referred to as DTC).
**Algorithm 1: Decision Tree Construction Algorithm (DTC)**1: Input: initial root node $x_{0}$. 2: Initialize parameters (θ,ω,λ), λ∈[0,1];3: For (n=0;n<N;n++) // N is the maximum number of iterations.4:       get π(x|a,θ); 5:       $x=x_{0}$;6:       while f(x)≠0
// f(x) = 0 if and only if x is a leaf node.7:           a=π(x|a,θ);
8:           x=NextNode(x,a);
// NextNode(x,a) is to select the next non-leaf node in depth-first traversal order.9:           NandA($x_{0}$)←x,a;// NandA($x_{0}$) is used to record all pairs (x,a).10:       End while11:       dθ=0,dω=0;
12:       get $V_{n}\left(x,\omega \right)$ according to formula (3);13:       For each$\left(x,a\right)\in \mathrm{NandA}\left(x_{0}\right)$
14:            get R from environment;15:            $d\omega =d\omega +\partial {\left(V_{n}\left(x,\omega \right)-R\right)}^{2}/\partial \omega$;16:            d$\theta =d\theta +(\partial (\pi (x|a,\theta )/\partial \theta )\cdot (\partial \left(V_{n}\left(x,\omega \right)-R\right)/\partial \pi (x|a,\theta ))$;17:       End for18:       θ=θ+dθ,ω=ω+dω;
19: End for20: Output: strategy π(x|a,θ), value function $V_{n}\left(x,\omega \right)$


The input of DTC is the initial root node. After initializing the parameters, in each round of iteration, by performing an action (given a node x∈X, get a division action a∈A according to the strategy) on each non-terminal leaf node (a terminal leaf node is a node whose number of rules is less than the given threshold, the threshold is described in Section 3.2) according to the current strategy π(x|a,θ), build the decision tree step by step in the depth-first traversal order. When the decision tree is constructed, R is the cumulative return obtained in the agent, $V_{n}\left(x,\omega \right)$ is the cumulative return in the sample. Adjust the model parameters by R and $V_{n}\left(x,\omega \right)$. After multiple rounds of iterations, finally determine the parameters θ and ω, output the strategy π(x|a,θ) and value function $V_{n}\left(x,\omega \right)$.

The flow of the rule matching algorithm based on heterogeneous integrated learning is shown in Algorithm 2 (hereinafter referred to as RMHIL).
**Algorithm 2: Rule Matching Algorithm based on Heterogeneous Integrated Learning (RMHIL)**1: Input: a flow packet and a set of rules2: Call Algorithm 1 (DTC) to get strategy
π (x|a,θ);3: Construct a decision tree of the rule set according to the optimal strategy;4: Traverse the decision tree to select the highest priority rule.5: Output: the rule matching the flow packet

### 3.2. Algorithm Implementation

The algorithm is implemented according to Algorithm 2. The algorithm implementation language is Python, and the editor used is pycharm-community-2019.1.1.

The important step in the implementation process is to determine the data structure of the rule and decision tree. We create class “rule”, class “node”, and class “decision tree”.

In class “rule”, we fix the rule fields and define priority and field value range. The field value range includes the value range of all rule fields. For example, if the rule field is the source/destination IP and the data input is 1.0.0.0/24, the calculation method of the field value range is to convert it to a 32-bit binary code and then reserve (32-24)-bit network bits. The left end of the value range is the value that all 24-bit host bits are 0. The right end of the value range is the value that all 24-bit host bits are 1; that is, the value range is [1.0.0.0, 1.255.255.255]. If the rule field is the source/destination port number and the data input is 20, the field value range is [20, 20]. If the rule field is protocol and the data input is 0x06/0xFF (representing protocol 06 TCP), the field value range is [06, 06].

In class “node”, we define attributes such as node ID, value range, including rules, depth in the tree, and children node. Given d rule fields, we use 4d values to encode the value range of a tree node, including the value range of the left and right branches of the node on each field. 

Since the tree depth can only be determined after the decision tree is constructed, we should consider how to encode the state of the decision tree with dynamic tree depth. A fixed-length state representation needs to be designed. Since the environment constructs a decision tree node by node, and the rules of each node are included in the rules of its parent node, we only need to encode the current tree node state and use it as the input of the agent.

In class “decision tree”, we define attributes such as rule set, root node, tree depth, node number, leaf node threshold, current node, and nodes to be divided.

In addition, the division action a is expressed as [division dimension, division value].

Introducing field value range and leaf node threshold alleviates the problem of rule overlap.

## 4. Comparative Experimental and Performance Verification

The rule matching algorithm we propose (RMHIL) and the existing efficient rule matching algorithms (HyperCuts [15] and CutTSS [18]) are based on decision trees. These three rule matching algorithms generate a decision tree from the rules and then traverse the decision tree to find the matching rules. In the rule matching algorithm based on decision trees, the quality of the constructed decision tree directly determines the performance of the algorithm. The better the performance of the decision tree building algorithm, the better the performance of the rule matching algorithm.

Therefore, we compare the performance of RMHIL, HyperCuts, and CutTSS by conducting comparative experiments on the decision tree construction algorithms of the three algorithms. The decision tree building algorithms of RMHIL are otherwise named DTC. For the convenience of description, the decision tree construction algorithms of the three algorithms are referred to as DTC, HyperCuts, and CutTSS.

The metrics used are matching time and memory overhead, which are the focus of general rule matching algorithms. Since we use the same underlying tree data structure for all algorithms, a lower tree depth can ensure faster matching. The shallower the tree, the fewer nodes need to go to find the matching rule, and the matching time is naturally shorter. So, we use the tree depth to measure the matching time. Similarly, we use a quotient to measure the memory overhead. This quotient is the result of dividing the number of rules for all nodes by the number of records in the rule set. The smaller the quotient is, the smaller the average number of repeated occurrences of each rule in the decision tree is, and the less memory is occupied. 

Generally, only the 5 fields of source IP, destination IP, source port number, destination port number, and protocol are matched in the rule. And the most widely used sample at present is the 5-field sample. So this section measures the matching time and memory usage on 5-field data sets of different magnitudes based on different values (0,1) of variable λ.

The rule data set used in the experiment is the public data set published in reference [25]. Since only 5 fields are used for rule matching, we do a simple preprocessing after importing the data set (only the required fields are used in the rule set). The experiment in this section uses a total of 30 data sets. The number of rules and their magnitudes (the number of digits to which the number of rules belongs) are shown in Table 2 (arranged in ascending order of the number of rules).

### 4.1. Matching Time

**Figure 4:**
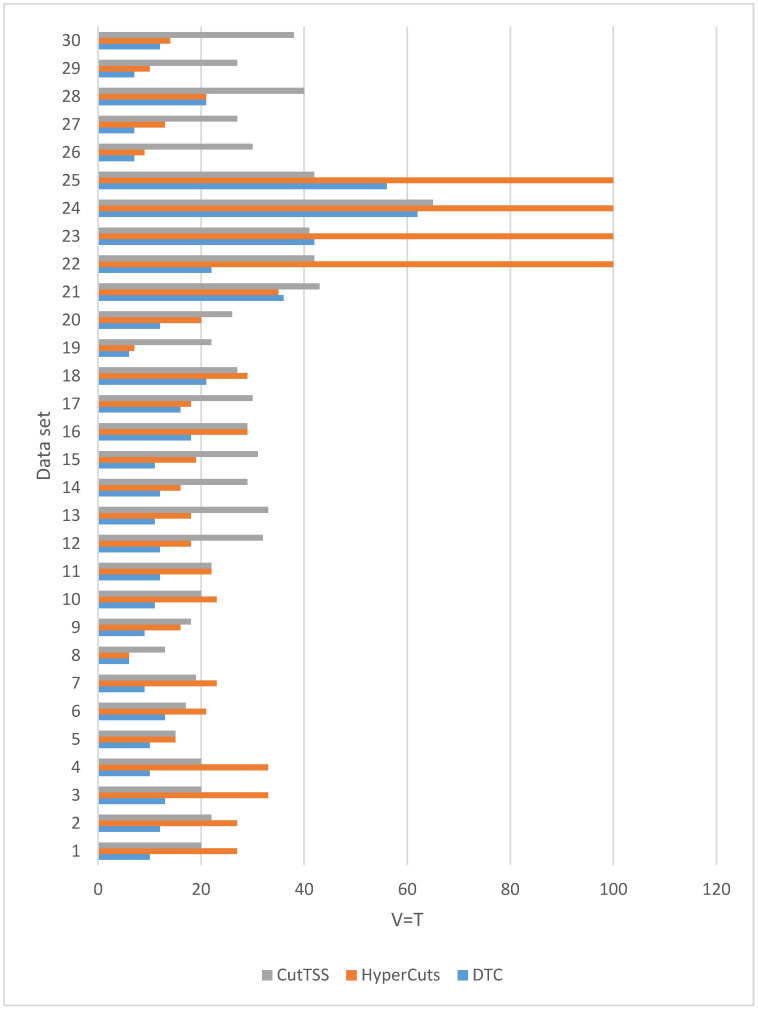
The experimental result when V = T (matching time).

When λ is 1, that is $V_{n}=T_{n}$, the experimental results of the three algorithms on different magnitude data sets are shown in Figure 4.

It can be seen from Figure 4 that the depth of the decision tree (representing matching time) constructed by DTC is not higher than that of the decision tree constructed by HyperCuts and CutTSS. Therefore, DTC has obvious advantages in matching time.

In order to better observe the experimental results, we analyze the experimental results separately from three magnitudes.

**Figure 5:**
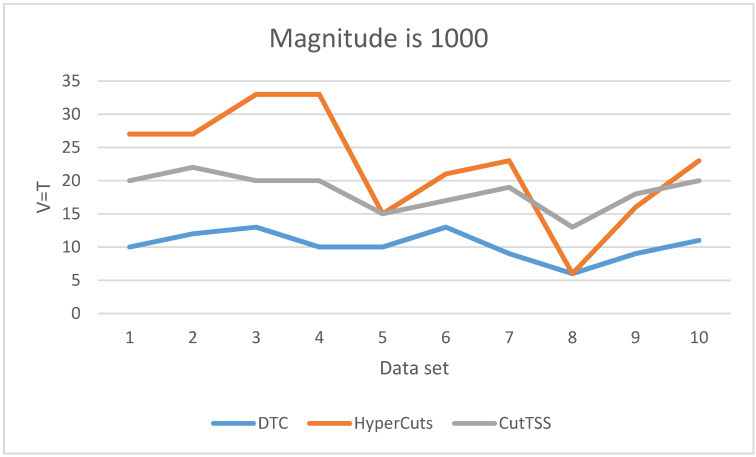
The experimental result when V = T and the data magnitude is 1000.

As is shown in Figure 5, when the magnitude of the data set is 1000, DTC has the least matching time and little fluctuation. The matching time of algorithm CutTSS does not fluctuate greatly and is shorter than algorithm HyperCuts in most cases. The matching time of algorithm HyperCuts fluctuates greatly and takes a long time. HyperCuts does not consider the problem of rule overlap, so its matching time is very easily affected by the content of the data set. The content of the data set varies for each test, resulting in different degrees of rule overlap. As a result, the loss time of HyperCuts is different, and eventually, the matching time of HyperCuts fluctuates greatly. Both DTC and CutTSS alleviate this problem, and the fluctuation of matching time is small.

**Figure 6:**
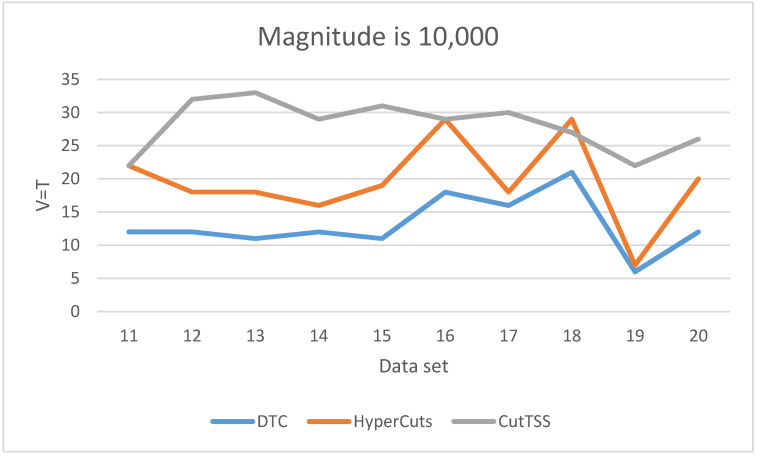
The experimental result when V = T and the data magnitude is 10,000.

As is shown in Figure 6, when the magnitude of the data set is 10,000, DTC has the least matching time and little fluctuation. The matching time of algorithm HyperCuts fluctuates greatly, but it is shorter than algorithm CutTSS in most cases. The matching time of algorithm CutTSS has no major fluctuations but is the longest. The fluctuation of the matching time of the three algorithms is consistent with the situation when the magnitude is 1000. We guess that the overlap problem in these data sets may be lighter, making the matching time of algorithm HyperCuts shorter.

**Figure 7:**
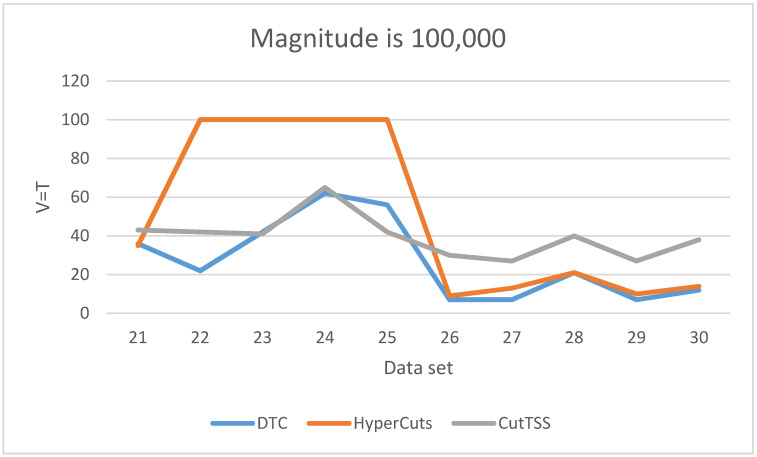
The experimental result when V = T and the data magnitude is 100,000.

As is shown in Figure 7, when the magnitude of the data set is 100,000, the matching time of DTC is the least and slightly fluctuates. The matching time of algorithm CutTSS does not fluctuate greatly and is longer than DTC. The matching time of algorithm HyperCuts fluctuates greatly. When the matching time is too long, we mark T = 100 and no longer test. By comparing the content of data set 22–25 with other data sets, we find that the port number of data set 22–25 has more field values, which confirms that the matching time of algorithm HyperCuts is very sensitive to the content of the data set. The disadvantage of algorithm HyperCuts is that it does not consider the problem of rule overlap appearing here.

Among the three algorithms, only the matching time of HyperCuts fluctuates greatly because only it does not consider the overlapping rule problem.

The above analysis shows that the matching time of DTC is shorter than that of algorithms HyperCuts and CutTSS. 

### 4.2. Memory Overhead

**Figure 8:**
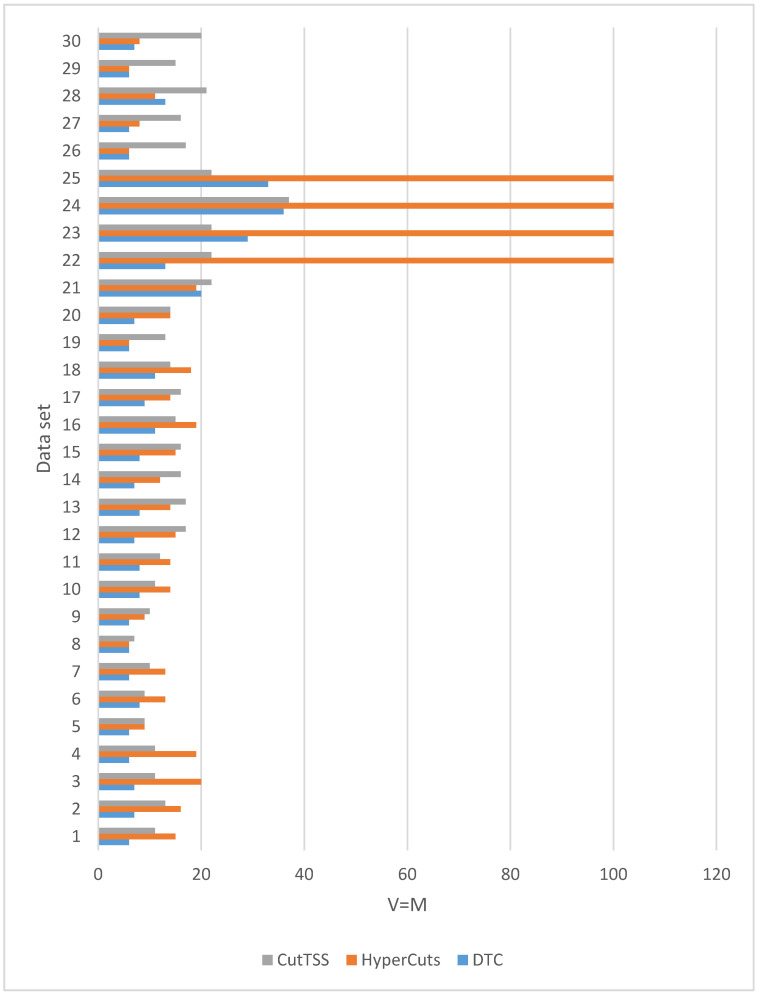
The experimental result when V = M (memory overhead).

When λ is 0, that is $V_{n}=M_{n}$, the experimental results of the three algorithms on different magnitude data sets are shown in Figure 8.

We have 30 data sets. It can be seen from Figure 8 that in 26 data sets, the memory overhead of the decision tree constructed by DTC is not more than that of algorithms HyperCuts and CutTSS. In 4 data sets (21, 23, 25, 28), the memory overhead of the decision tree constructed by DTC is slightly more than that of the algorithms HyperCuts or CutTSS. Generally speaking, compared with the algorithms HyperCuts and CutTSS, DTC has a slight advantage in memory overhead.

In summary, compared with algorithms HyperCuts and CutTSS, DTC has obvious and stable advantages in matching time and has a slight advantage in memory overhead. Therefore, RMHIL, which uses DTC, has advantages in matching time and memory overhead.

## 5. Conclusions

Based on heterogeneous integrated learning, we solve the rule matching problem of SDN flow forwarding. We describe and transform the problem. We design and implement RMHIL, a rule matching algorithm based on reinforcement learning, recurrent neural network, and decision tree. Through comparative experiments with the algorithms HyperCuts and CutTSS, it is shown that RMHIL has advantages in matching time and memory overhead.

During the experiment, we find that there are still shortcomings, which will be further improved in the follow-up work:

(1) With the continuous expansion of network scale and the continuous increase of SDN functional requirements, the most commonly used 5-field matching method will be difficult to meet the demand. Therefore, we must consider the 40-field rule matching. Although the sample construction method is simple and the software is ready, the strategy generation and value function calculation of a large number of 40-field real samples take a lot of time. It is planned to be completed in the next step, and then the algorithm performance test is performed based on the sample.
(2) The algorithm proposed in this paper does not have obvious advantages in memory usage. We consider fusing as the means of reducing the rule overlap problem in the CutTSS algorithm (first dividing the rule set and then constructing the decision tree in parallel) or designing efficient and feasible solutions for the characteristics of the algorithm.
(3) We do not use the countdown in the traditional sense when measuring the matching time.
(4) A custom standard is used to facilitate comparative experiments. To further optimize the matching time of the algorithm proposed in this paper, we consider using GPU version TensorFlow, AWS cloud computing service platform, and other acceleration methods.

In addition, our proposed rule matching method applies not only to SDN but also to sensor networks. In SDN, a data transmission path must be established between the source switch and the destination switch. After receiving the data packet transmitted by the source switch, the intermediate node checks whether it matches an entry in the current rule set. The implementation environment of RMHIL in SDN is an OpenFlow switch, the input is a data packet and a rule set, and the output is an entry that matches the data packet in the rule set. In a sensor network, a data transmission path must be established between the source sensor node and the sink node. After receiving the interest information published by the sink node, the intermediate nodes check whether it matches an entry in the local interest list. The implementation environment of RMHIL in the sensor network is the sensor node, the input is the interest information and the interest list, and the output is an entry in the interest list that matches the interest information. Therefore, it is convenient and easy to apply RMHIL to sensor networks.

## Figures and Tables

**Table 1:** This is a simple example of rule set, where * represents a null value.

Source IP/IPv6 Address	Destination IP/IPv6 Address	TCP/UDP Source Port Number	TCP/UDP Destination Port Number	Protocol	Instructions Set	Counters	Priority	Timeout	Cookie
1.0.0.0/32	1.0.1.0/32	*	*	TCP	drop	3	0	00:50	A
*	*	0	20	TCP	add	5	1	00:39	B
*	*	*	*	TCP	change	4	2	00:27	C

**Table 2:** The information of the rule data set.

Data Set	The Number Of Rules	Magnitude	Data Set	The Number of Rules	Magnitude	Data Set	The Number of Rules	Magnitude
1	799	1000	11	7298	10,000	21	75,100	100,000
2	846	1000	12	8772	10,000	22	83,752	100,000
3	857	1000	13	8833	10,000	23	83,797	100,000
4	863	1000	14	9039	10,000	24	83,966	100,000
5	934	1000	15	9377	10,000	25	88,081	100,000
6	942	1000	16	9418	10,000	26	96,078	100,000
7	961	1000	17	9476	10,000	27	98,180	100,000
8	970	1000	18	9591	10,000	28	99,052	100,000
9	990	1000	19	9655	10,000	29	99,318	100,000
10	990	1000	20	9774	10,000	30	99,480	100,000

## Data Availability

Data is contained within the article. The data presented in this study are available in article.
